# Neurologic Music Therapy via Telehealth: A Survey of Clinician Experiences, Trends, and Recommendations During the COVID-19 Pandemic

**DOI:** 10.3389/fnins.2021.648489

**Published:** 2021-04-08

**Authors:** Lauren Patricia Cole, Tara Lynn Henechowicz, Kyurim Kang, Marija Pranjić, Nicole Marie Richard, Gloria L. J. Tian, Corene Hurt-Thaut

**Affiliations:** Music and Health Science Research Collaboratory, Faculty of Music, University of Toronto, Toronto, ON, Canada

**Keywords:** telehealth, neurologic music therapy, music therapy, pandemic, COVID-19, music, rhythmic auditory stimulation, rehabilitation

## Abstract

This cross-sectional survey investigated the transition of Neurologic Music Therapy (NMT) services from in-person (pre-COVID-19) to telehealth (since COVID-19) to (1) determine whether the use of an NMT paradigm contributes to the successful transition of therapy services to telehealth, (2) identify which NMT domains and techniques are transferable from in-person to telehealth, (3) identify whether there are differences in the transition of NMT services across different employment settings, and (4) evaluate the potential benefits and challenges of telehealth NMT. An online survey comprised of 49 closed and open-ended questions was distributed by the Academy of Neurologic Music Therapy to 2,778 NMT affiliates worldwide. The survey sought information on demographics, telehealth perceptions, technology, assessment, clinical practice, safety, and caregiver involvement. Quantitative and qualitative analyses were applied. Eighty-one participants answered the survey and the 69 who completed the survey in its entirety were included in the analysis. Results indicated that the frequency of NMT technique usage had no impact on the overall number of clinical hours retained over telehealth. Correlation analysis revealed an association between more frequent NMT usage and perceived likelihood of using telehealth in the future (i.e., once COVID-19 is no longer a major threat), as well as with fewer group sessions lost over telehealth. All NMT domains transferred to telehealth, although within the sensorimotor domain, fewer therapists implemented rhythmic auditory stimulation for telehealth sessions compared to in-person. Overall, NMTs had fewer hours for telehealth compared to in-person regardless of employment setting. Technological challenges were notable drawbacks, while major benefits included the ability to continue providing NMT when in-person sessions were not possible, increased accessibility for remote clients, and positive outcomes related to increased caregiver involvement. Based on the results, our recommendations for implementing telehealth in Neurologic Music Therapy include integrating telehealth into routine care, mitigating safety concerns, identifying those who could benefit most from remote delivery, involving caregivers, and developing/sharing resources for telehealth NMT.

## Introduction

Music has the power to bolster emotional health, provide a sense of interpersonal connection, and is used as a therapeutic modality (Huron, 2001; Stupacher et al., 2017). Musical performances on balconies and live-streams became prominent beacons of unity during the coronavirus disease 2019 (COVID-19) pandemic. The COVID-19 pandemic disrupted lives worldwide, taking a toll on mental and physical health across the life span and challenging health care systems in countless ways (Brooks et al., 2020; Fiorillo and Gorwood, 2020; Holmes et al., 2020). The need for physical distancing forced health care providers to rapidly implement telehealth services to continue to provide needed medical care (Smith et al., 2020; Wosik et al., 2020). The term ‘telehealth’ refers to the distribution of health-related services at a distance via remote technologies (Wosik et al., 2020). Due to its online and distanced format, telehealth is therefore well suited for public health emergencies such as pandemics (Lurie and Carr, 2018; Hollander and Carr, 2020). Neurologic music therapists (NMTs) adapted and shifted their in-person services to online platforms to continue providing therapy services and meet the emerging needs of vulnerable individuals.

Neurologic Music Therapy (NMT) is an evidence-based treatment model in which standardized therapeutic music interventions address sensorimotor, cognitive, and speech and language dysfunctions arising from neurological disease and impairments, as well as the psychosocial needs of a client (Thaut and Hoemberg, 2014). NMT is based on the neuroscientific relationship between music perception, cognition, and production and is used to facilitate functional change. It is distinguished from traditional music therapy which has historically focused on a social science model of well-being, relationship building, and emotional support based on cultural, interpretative, and associative roles of music (Thaut and Hoemberg, 2014). The regulation of traditional music therapy varies between national jurisdictions with different training requirements and regulatory bodies. Though there is a tremendous amount of valuable work done by non-NMT music therapists, the varying theoretical backgrounds and training can lead to disparity in types of interventions used from one music therapist to the next to address similar clinical goals. The Academy of NMT provides comprehensive training and regulation of NMT global practice. Since NMT clinical practice is supported by empirical evidence and consistent across the world, NMT is endorsed by organizations including the World Federation of Neurologic Rehabilitation, the European Federation of Neurorehabilitation Societies, and the International Society for Clinical Neuromusicology (The Academy of Neurologic Music Therapy, 2020).

Several studies published prior to the pandemic investigated telehealth delivery of music therapy in general (i.e., not specifically NMT), across a variety of clinical settings. Music therapy students and supervisors at the Berklee School of Music delivered music therapy services to a school for child soldiers in Lira, Uganda. Various forms of music making were used, including singing, dancing, call-and-response drumming, and improvised music (Vaudreuil et al., 2020). A case-study by Lightstone et al. (2015) reported that telehealth music therapy (led by a music therapist together with a clinical psychologist) was effective in addressing post-traumatic stress disorder and major depressive disorder in a veteran, evidenced by the client’s retrospective self-report. Interventions reported by Lightstone et al. (2015) involved musical improvisation of various emotions to improve the client’s ability to tolerate, express, and avoid dissociation related to different emotions, followed by discussion of these improvisations with the clinical psychologist. Vaudreuil et al. (2020) reviewed several published and unpublished examples of music therapy and telehealth, relating to sessions with veterans. They reported that music therapy and other creative arts therapies can be promising avenues of holistic treatment for veterans whose physical location made it difficult for them to access in-person services. A case study involving an autistic adolescent conducted by Baker and Krout (2009) found that using telehealth to work on a songwriting intervention yielded more engagement and appropriate social interaction from the client including increased eye contact, laughing, and openness, when compared to an in-person format. Finally, Fuller and McLeod (2019) used telehealth music therapy to work with children with hearing loss and their parents. They observed that family tele-intervention provided a high level of parent-child interaction, and parent skill development. These case studies and single-project studies are compelling, as they provide preliminary evidence that telehealth music therapy may be as beneficial as in-person therapy in certain situations (Baker and Krout, 2009); that telehealth may reach clients who would not ordinarily be able to access services (Lightstone et al., 2015; Vaudreuil et al., 2020); or that telehealth may help to address client needs in a more robust manner by involving caregivers in sessions (Fuller and McLeod, 2019). However, in addition to the limitations of case studies, generalizability is also limited due to variability of music therapy techniques.

The increased scope of telehealth music therapy since the onset of the pandemic demands new research to ascertain wider patterns of benefits, challenges, and perceptions of telehealth for a variety of client areas. To our knowledge, only one study has been published on telehealth music therapy since the onset of the pandemic. Gaddy et al. (2020) found that music therapists in the United States of America experienced job stresses, including fewer client hours and an increase in the use of telehealth modality. They reported that music therapists experienced a moderate level of stress, but higher hope than the general (United States) population, according to the Adult Hope Scale and the Perceived Stress Scale-10. Although this study provided trends relating to telehealth in music therapy, it did not primarily address whether music therapists had success continuing to see their clients over telehealth, or what factors led to their success. Moreover, focus was put on the general music therapy population rather than NMT specifically, which is the population of interest in the current study.

In response to the pandemic, the Academy of Neurologic Music Therapy facilitated monthly global online meetings to support NMTs navigating the transition to telehealth. The researchers in the current study attended these meetings and noted anecdotes regarding telehealth’s benefits, challenges, and strategies for navigating difficulties. Out of these meetings emerged a need to systematically examine which factors allow for successful transitions from in-person to telehealth practice and identify the main benefits and drawbacks of telehealth models to develop guidelines for immediate implementation during the pandemic and onwards. To address the described gaps in the literature, our survey study targeted the following research questions: (1) Did therapists using NMT techniques more frequently retain more clinical hours in the transition to telehealth compared to those who used NMT less frequently? (2) Did NMT domains and techniques change for telehealth compared to in-person delivery? (3) Did employment setting influence how clinical hours were affected by transition from in-person to telehealth? (4) What were NMT clinicians’ perceptions of benefits and disadvantages of a telehealth model of NMT?

## Materials and Methods

### Materials

The researchers designed a 49-question survey divided into seven sections (Demographics, Perceptions, Technology, Assessments, Clinical Practice, Safety, and Caregiver Involvement). Survey questions asked participants about their experience delivering services for in-person versus telehealth. The survey was piloted with five NMTs who were not directly involved in the study, and their feedback was incorporated into the final survey design. The survey was hosted on the online survey platform Survey Monkey. The survey included 44 closed and five open-ended questions, those marked with an asterisk (^∗^) were mandatory (see Supplementary Material).

### Data Collection

An invitation email containing a link to the online survey was sent to the 2,778 affiliates of the Academy of Neurologic Music Therapy. Links to the invitation letter and the survey were also posted on closed Facebook sites for NMT members. NMT members included music therapists, music therapy students, and other rehabilitation professionals (e.g., occupational therapists, physiotherapists, and speech therapists) from 57 countries, who have taken the 4-day NMT training. The period of data collection spanned from September 9th to 25th, 2020.

The inclusion criteria for NMTs to participate in this survey were to (1) read and understand English; (2) be over the age of 18; (3) be an active NMT Academy Affiliate; (4) have practiced music therapy (including NMT or other techniques) over telehealth with at least one client and/or group; and (5) be accredited to practice music therapy in their region. Participants could withdraw from the survey at any time without any penalty. Participants consented to participate in the study and confirmed that they met the inclusion criteria, prior to completing the survey. The Research Ethics Boards of the University of Toronto approved the study protocol.

### Data Analysis

During the data collection period, 81 participants responded to the survey. Eleven participants were excluded from the analysis as they discontinued the survey before completing Question #35: “How frequently do you use NMT?”.

Although participants confirmed their eligibility for the study at the beginning of the survey, additional screening was performed on NMT designation and professional credentials via the first survey questions. One participant was removed because they did not have an NMT designation. As a result, a total of 69 participants were included in this analysis. The number of participants included in each analysis differed as for some questions, responses were not mandatory.

#### Quantitative Analysis

All statistical analyses were conducted in R (R Core Team, 2019). Results were determined significant at the alpha = 0.05 level.

##### Frequent-NMT clinicians vs. non-frequent-NMT clinicians on clinical hours

A two-way mixed ANOVA was conducted to investigate if there was a significant difference between frequent NMT and non-frequent NMT users (between subjects) and session delivery methods (within subjects: in-person and telehealth) on clinical hours. Significant effects were further investigated with pairwise *t*-tests. *P*-values were adjusted using the Bonferroni correction method. It is important to note that we have excluded one participant from this analysis due to an error in data collection. Due to an error on the survey which allowed participants to select up to 40 clinical hours for in-person sessions prior to the transition to telehealth (see Question 11) and up to 100 hours for sessions via telehealth since the transition (Question 12), one data point could not be interpreted and was removed. All other data points ≤ 40 were included in the analysis.

##### NMT domains and technique applications during in-person and telehealth sessions

A chi-square test of independence was carried out to determine if there was a relationship between session delivery methods (in-person and telehealth) and the total implementation of NMT for three domains (sensorimotor, cognitive, and speech/language). Within each domain, another chi-square test was computed to assess whether there were relationships between session delivery methods (in-person and telehealth) and the number of clinicians using NMT techniques.

Sensorimotor NMT techniques included rhythmic auditory stimulation (RAS), therapeutic instrumental music performance, and patterned sensory enhancement. Cognitive NMT techniques included musical attention control training, musical executive function training, associative mood and memory training, musical sensory orientation training, musical echoic memory training, auditory perception training, auditory perception training sensory integration, musical mnemonics training, musical neglect training, and music in psychosocial training and counseling. Lastly, speech and language NMT techniques included developmental speech and language training through music, musical speech stimulation, symbolic communication training through music, musical intonation therapy, rhythmic speech cueing, oral motor and respiratory exercises, vocal intonation therapy, and therapeutic singing (Thaut and Hoemberg, 2014).

##### Influence of employment settings on in-person and telehealth sessions

To analyze the influence of employment settings, participants were grouped into three categories: employed by a facility, employed/contracted by a private practice, and owned/ran a private practice. NMTs in each of these settings would have different factors influencing their job security, such as source of client referrals and payment for services. One participant was excluded due to the same reason in the section: “Frequent-NMT Clinicians vs. Non-frequent-NMT Clinicians on Clinical Hours.”

A two-way mixed ANOVA was carried out to investigate if there was a significant difference between employment settings (between subjects) and session delivery methods (in-person and telehealth, within subjects) on clinical hours. Significant effects were further determined with pairwise *t*-tests. *P*-values were adjusted using the Bonferroni correction method.

##### Correlation analysis

For more detailed relationships between variables, Spearman’s rank-order correlations were computed. A total of six variables were included (1) the frequency of using NMT in telehealth (NMT-telehealth), (2) number of new individual clients since transitioning to telehealth, (3) discontinued individual clients since transitioning to telehealth, (4) discontinued group clients since transitioning to telehealth, (5) future telehealth (“Do you see yourself continuing to use telehealth in the future [i.e., after COVID is no longer a major threat]?”), and (6) perception of NMT designation (“The NMT designation has been beneficial and/or had a direct positive impact on my ability to do telehealth”).

#### Qualitative Analysis

A conventional Qualitative Content Analysis (QCA) approach was applied to open-ended responses. Conventional QCA is used to help describe a phenomenon which is novel or has no theory to yet describe it (Hsieh and Shannon, 2005), such as perceptions of the benefits and challenges of telehealth among music therapists. QCA is widely used in health care research and can help to identify objective patterns and themes present across individuals’ subjective experiences (Elo and Kyngäs, 2008).

Survey questions analyzed using QCA included the following: “Please list any of the benefits that telehealth provides,” “Please list any of the drawbacks to telehealth,” “Briefly list the main technical difficulties you experienced,” “Have you addressed goal areas related to stresses, isolation, disruption to everyday routines, and other challenges caused by the pandemic? Please explain,” and “Are there any NMT techniques that you have client safety concerns for administering through telehealth? Explain your concerns.”

For the conventional QCA process, two researchers independently read participants’ open-ended responses, and summarized each statement based on themes that emerged. Raters then compared their summaries and created a finite number of categories corresponding to the most common themes for each question. The two researchers independently coded responses numerically according to the corresponding category. Where the two raters differed on their coding, they discussed the original text in the response to determine what would be the best coding until they could agree on which salient themes were present. The frequencies of each theme presented were tallied to identify the most common themes for each question.

## Results

### Participants

#### Demographics

The participants included 69 NMTs, a response rate of ∼3%. The mean age (±standard deviation) of participants was 39.0 ± 11.3 years. Respondents primarily practiced NMT in the United States (*n* = 47) and in Canada (*n* = 8). The mean years of practicing NMT was 5.9 ± 5.3 years, and the mean years of practicing professional services (e.g., music therapy or physiotherapy) was 11.7 ± 10.0 years. Ninety percent of participants identified as female (*n* = 62). Fifty percent of participants reported having a full case load (*n* = 34).

Regarding clinical populations and employment settings, the participants reported working with children (*n* = 52), adults (*n* = 52), adolescents (*n* = 44), and older adults (*n* = 42). Most NMTs worked in a private practice setting (*n* = 38) followed by in an institute (*n* = 29). Three NMTs were retired or unemployed. Most NMTs worked with clients in neurodevelopmental populations (*n* = 48), followed by chronic neurorehabilitation (*n* = 37), and geriatrics (*n* = 28). The demographic information of the participants is summarized in Table 1.

**TABLE 1:** Demographic data for neurologic music therapists (*N* = 69).

Variables	Mean ± SD
Age	39.0 ± 11.3
Practice professional service years	11.7 ± 10.0
Practice NMT years	5.9 ± 5.3

	**# of clinicians (%)**

**Sex**	
Male	6 (8.7)
Female	62 (89.9)
Non-binary/third gender	1 (1.4)
**Education**	
Bachelor’s degree	29 (42.0)
Master’s degree	34 (49.3)
Ph.D. degree	2 (2.9)
Other	4 (5.8)
**Country**	
United States	47 (68.1)
Canada	8 (11.6)
United Kingdom	3 (4.3)
Netherlands	2 (2.9)
Germany	1 (1.4)
Ireland	1 (1.4)
Australia	2 (2.9)
South Africa	1 (1.4)
Taiwan	1 (1.4)
Singapore	1 (1.4)
Argentina	2 (2.9)

	**# of clinicians**

**Client populations**	
Neurodevelopmental populations	48
Chronic neurorehabilitation	37
Geriatric care and dementia	28
Acute neurorehabilitation	24
Mental health/Psychiatric	21
Adapted music education	16
End of life/palliative	14
Physical rehabilitation	12
Neonatal care	3
**Client age groups**	
Children	52
Adolescents	44
Adults	52
Older adults	42

#### Participants’ Telehealth Experiences

Prior to COVID-19, most participants conducted sessions in-person (*n* = 64), five participants conducted sessions both in-person and via telehealth, and no participants used telehealth as the sole method of delivery. Since adjusting to the constraints of the COVID-19 pandemic, most participants conducted sessions through both in-person and telehealth (*n* = 50), many participants conducted sessions via telehealth (*n* = 16), and three participants worked with clients in-person.

All participants reported using Zoom for at least some telehealth sessions (*n* = 69). Additional telehealth platforms included doxy.me (*n* = 17), FaceTime (*n* = 18), Google Hangouts (*n* = 16), telephone (*n* = 7), WhatsApp video (*n* = 5), Microsoft Teams (*n* = 5), Skype (*n* = 3), Facebook video (*n* = 2), Google Meet (*n* = 2). Eight participants reported using other platforms.

Participants reported accessing various forms of assistance for the transition to telehealth. These included informal conversations with colleagues (*n* = 50), the NMT Global Support Meetings (*n* = 30), local NMT support chapter meetings (*n* = 18), and help from facilities (*n* = 32). Eighteen respondents indicated that they accessed other forms of assistance such as regional music therapy meetings and workshops, or their own personal resources and creativity (i.e., no external support).

### Quantitative Analysis Results

#### Frequent-NMT Clinicians vs. Non-frequent-NMT Clinicians on Clinical Hours

There was no significant interaction effect between frequent NMT/non-frequent NMT clinicians and session delivery methods on clinical hours, *F*(1,65) = 1.45, *p* = 0.23, and no significant main effect of frequent NMT/non-frequent NMT clinicians on clinical hours, *F*(1,65) = 3.93 *p* = 0.05. However, there was a significant main effect of session delivery methods on clinical hours, *F*(1,65) = 32.67, *p* < 0.001, indicating that clinical hours were significantly greater for in-person sessions (*M* = 21.40, *SD* = 12.16) compared to telehealth sessions (*M* = 15.96, *SD* = 12.36), *t*(66) = 5.57, *p.adj* ≤ 0.001.

#### NMT Technique Applications During In-Person and Telehealth Sessions

Table 2 shows the break-down of implementation of NMT techniques within each domain for in-person and telehealth. The sum of each row in Table 2 was extracted to calculate the total techniques implemented for NMT domains (cells in Table 3). Table 3 indicates the total techniques implemented for NMT domains (sensorimotor, cognition, and speech and language) by in-person and telehealth. There was no significant relationship between session delivery method and the distribution of total number implementation of NMT techniques in NMT domains, χ^2^(2) = 1.76, *p* = 0.42 (see Table 3). However, there was a significant relationship between session delivery method (telehealth and in-person) and the distribution of sensorimotor technique usage, χ^2^(2) = 6.08, *p* = 0.048 (see Table 2). For the cognitive and speech/language domains, there were no significant relationships between session delivery methods and the distribution of techniques (see Table 2).

**TABLE 2:** Breakdown of implementation of NMT by technique.

*Sensorimotor*				
			RAS		PSE			TIMP			Total
In-person			39 (76%)		49 (58%)			55 (57%)			143 (62%)
Telehealth			12 (24%)		36 (42%)			41 (43%)			89 (38%)

***Cognition***											

	**MACT**	**MEFT**	**AMMT**	**MSOT**	**MEM**	**APT**	**APTSI**	**MMT**	**MNT**	**MPC**	**Total**

In-person	51 (55%)	44 (53%)	26 (60%)	34 (63%)	19 (59%)	31 (57%)	26 (59%)	24 (59%)	13 (81%)	37 (50%)	305 (57%)
Telehealth	42 (45%)	39 (47%)	17 (40%)	20 (37%)	13 (41%)	23 (43%)	18 (41%)	17 (41%)	3 (19%)	37 (50%)	229 (43%)
Total	93 (100%)	83 (100%)	43 (100%)	54 (100%)	32 (100%)	54 (100%)	44 (100%)	41 (100%)	16 (100%)	74 (100%)	534 (100%)

***Speech and language***									

	**DSLM**	**MUSTIM**		**SYCOM**		**MIT**	**RSC**	**ORMEX**	**VIT**	**TS**	**Total**

In-person	39 (54%)	35		26		26 (60%)	39 (58%)	34	24 (55%)	49 (52%)	272 (57%)
		(57%)		(67%)				(58%)			
Telehealth	33 (56%)	26		13		17 (40%)	28 (42%)	25	20 (45%)	46 (48%)	208 (43%)
		(43%)	(33%)					(42%)			
Total	72 (100%)	61		39		43 (100%)	67 (100%)	59	44 (100%)	95 (100%)	480 (100%)
		(100%)		(100%)				(100%)			

**RAS, rhythmic auditory stimulation; PSE, patterned sensory enhancement; TIMP, therapeutical instrumental music performance; MACT, musical attention control training; MEFT, musical executive function training; AMMT, associative mood and memory training; MSOT, musical sensory orientation training; MEM, musical echoic memory training; APT, auditory perception training; APTSI, auditory perception training sensory integration; MMT, musical mnemonics training; MNT, musical neglect training; MPC, music in psychosocial training and counseling; DSLM, developmental speech and language training through music; MUSTIM, musical speech stimulation; SYCOM, symbolic communication training through Music; MIT, musical intonation therapy; RSC, rhythmic speech cueing; OMREX, oral motor and respiratory exercises; VIT, vocal intonation therapy; TS, therapeutic singing*.*

**TABLE 3:** Implementation of NMT by domain for in-person versus telehealth.

	Motor	Cognition	Speech/language	Total
In-person	143 (20%)	305 (42%)	272 (38%)	720 (100%)
Telehealth	89 (17%)	229 (43%)	208 (40%)	526 (100%)
% decrease	54 (38%)	76 (25%)	64 (24%)	194 (27%)

#### Influence of Employment Setting on In-Person and Telehealth Sessions

There was no significant interaction effect between employment settings and session delivery methods on clinical hours, *F*(2,62) = 0.45, *p* = 0.64. There was no significant main effect of employment setting on clinical hours, *F*(2,62) = 2.40, *p* = 0.10.

There was a significant main effect of session delivery methods on clinical hours, *F*(1,62) = 28.40, *p* < 0.001, indicating significantly more clinical hours for in-person sessions (*M* = 21.55, *SD* = 12.22) compared to telehealth sessions (*M* = 16.20, *SD* = 12.46), *t*(64) = −5.37, *p.adj* < 0.001.

#### Correlations

Table 4 indicates the correlations between six variables. There was a significant positive correlation between the frequency of NMT-telehealth and future telehealth, *r*_*s*_(4) = 0.30, *p* = 0.03. In other words, using NMT techniques more frequently in telehealth sessions was associated with one’s perceived likelihood of using telehealth in the future. Accordingly, the number of new individual clients positively correlated with future telehealth, *r*_*s*_(4) = 0.37, *p* < 0.01. That is, NMT clinicians who gained more new clients since transitioning to telehealth also had a higher perceived likelihood of using telehealth in the future.

**TABLE 4:** Correlations analysis table.

	1	2	3	4	5	6
(1) Frequency of NMT-telehealth	1					
(2) New client	0.14	1				
	(0.28)					
(3) Discontinued-individual	–0.22	–0.02	1			
	(0.09)	(0.90)				
(4) Discontinued-group	–0.29	0.15	0.42	1		
	(0.03)*	(0.24)	(0.00099)***			
(5) Future telehealth	0.30	0.37	–0.24	–0.07	1	
	(0.02)*	(0.004)**	(0.07)	(0.61)		
(6) Perceptions of NMT designation	0.39	0.03	–0.01	–0.04	0.09	1
	(0.002)**	(0.85)	(0.97)	(0.75)	(0.48)	

*p < 0.05, **p < 0.01, ***p < 0.001.

The frequency of NMT-telehealth was negatively correlated with the number of discontinued groups sessions, *r*_*s*_(4) = −0.29, *p* = 0.03. This indicates that clinicians who used NMT more frequently for telehealth sessions had fewer discontinued group sessions. The number of discontinued individual sessions was correlated with the number of discontinued group sessions, *r*_*s*_(4) = 0.42, *p* < 0.001. Lastly, perception of NMT designation was positively correlated with the frequency of NMT-telehealth, *r*_*s*_(4) = 0.39, *p* < 0.01. That is, NMTs who perceived an NMT designation as beneficial for their ability to do telehealth used NMT more frequently for telehealth sessions.

### Qualitative Analysis Results

Open-ended survey questions were related to five central themes: safety concerns, pandemic-related goals, benefits of telehealth, drawbacks to telehealth, and technology issues. Within each theme, several categories emerged from participants’ open-ended responses. Regarding safety, we asked: “Are there any NMT techniques that you have client safety concerns for administering through telehealth? Explain your concerns.” (see Table 5). Regarding pandemic-related goals, we asked: “Have you addressed goal areas related to stresses, isolation, disruption to everyday routines, and other challenges caused by the pandemic? Please explain.” (see Table 6). Regarding benefits, we asked respondents to “Please list any benefits that telehealth provides.” (see Table 7). Regarding drawbacks, we asked respondents to “Please list any of the drawbacks to telehealth.” (see Table 8). Any technological issues mentioned in response to the question about drawbacks were grouped into one category as our last theme was focused specifically on technical difficulties. To this end, we asked respondents to “Briefly list the main technical difficulties you experienced.” (see Table 9).

**TABLE 5:** NMT techniques posing safety concerns.

Category	No. (%)	Example quotations
None	22 (31.9)	“Nothing I use causes any concern;”
		“No - not in my work.”
None because caregiver is	12 (17.4)	“Training carers ensured safe delivery;”
present		“Not with my population and having caregiver involved;”
		“None if adequate support and supervision is available.”
RAS	21 (30.4)	“RAS gait due to not being present in-person for safety;”
		“RAS cannot use. The assessment and exercises are not available without physical support;”
		“Gait training if a caregiver is not present.”
Therapeutic instrumental music	2 (2.9)	“TIMP - motor concerns;”
performance (TIMP)		“Some PSE and TIMP exercises, especially with a client diagnosed with Quadriplegia due to CP.”
Patterned sensory	4 (5.8)	“PSE when the patient have (*sic*) balance issues;”
enhancement (PSE)		“Some PSE and TIMP exercises, especially with a client diagnosed with Quadriplegia due to CP.”
All motor techniques	6 (8.7)	“I have only been NMT since February, and I only use the physical rehabilitation interventions in person. I don’t feel confident to do them online;”
		“Motor techniques may pose safety concerns as I am not with the client to observe and physically support.”
Non-motor techniques	3 (4.3)	“[Musical Neglect Training]. Have been contacted about working with a client who had a major stroke and now has visual neglect. Concerned about safety considerations with regard to set up and implementation on her part without any in person support.”
		“[Music in Psychosocial Training and Counseling] can also be a concern if technical issues result in the session being interrupted or ended abruptly.”

**TABLE 6:** New goals related to pandemic.

Category	No. (%)	Example quotations
No: have not changed goals	17 (24.6)	“No. Have continued with same recognizable engagements in effort to provide familiarity and normalcy;”
		“Not in formal intervention, however, discussed with client/caregivers at end or beginning of sessions if topic arises;”
		“Not specifically.”
Yes: addressing general	17 (24.6)	“Yes, it is a major focus of my work and the reason for behind my funding;”
pandemic-related stresses		“I’ve been finding myself meeting the needs of my clients in the moment and focusing more on that with their written goals and objectives coming second;”
		“[Music in Psychosocial Training and Counselling] was used in a group setting, to help clients with autism to process current life event changes.”
Addressing anxiety	14 (20.2)	“Most of these goals are related to stress or anxiety around changes caused by the pandemic;”
		“Yes due to increased anxiety and pain;”
		“Stress release and stress management, as well as anxiety management.”
Addressing depression	5 (7.2)	“Addressing low mood;”
		“Stress and depression;”
		“Dealing with depression/isolation in elders.”
Increasing coping with wearing	2 (2.9)	“Preparation for mask wearing;”
a mask		“Coping with wearing a mask.”
Addressing social goals	6 (8.7)	“There has also been an increase in social goals and needs due to decreased social interactions;”
		“Goals around coping with social isolation and stress related to missing school/friends;”
		“But almost all groups and clients are suffering from isolation and stress.”
Increased assistance with	9 (13.0)	“I increased the amount of relaxation sessions;”
regulation		“Calming strategies;”
		“Increase in coping skills.”
Addressing disruption in	7 (10.1)	“Especially goals related to the disruption to everyday routines;”
routines		“I have a group that frequently process through their feelings of isolation and change in routine;”
		“Normalizing environment and establishing routine in a changed schedule.”
Addressing loss/trauma	3 (4.3)	“Also have addressed students and their families with members who have been sick with Covid-19 and were upset/stressed/grieving;”
		“I’ve been using lots of grounding and mindfulness techniques with my only telehealth client who also lost his grandmother.”

**TABLE 7:** Benefits of telehealth.

Category	No. (%)	Example quotations
Continuation of sessions	25 (36.2)	“Telehealth has allowed music therapy interventions to continue safely during the state of emergency;”
		“Continuity of care for patients;”
		“Able to maintain skills of clients with whom I work.”
Increased caregiver	15 (21.7)	“More family/caregiver participation and follow through;”
involvement		“Telehealth has allowed me to foster stronger relationships with my students’ families, which has facilitated collaboration and teaming with the family for each student and expanded the scope of practice.”
Increased accessibility	26 (37.7)	“Increased accessibility for clients who do not live close to a therapist;”
		“Accessibility to patients who do not have access to a facility or transportation;”
		“Solve the long distance problem.”
Comfort of being at home	10 (15.7)	“Clients appear more comfortable in a familiar space at home;”
		“Increased observation of client in natural setting;”
		“Ability to work from home.”
Safety from COVID-19	7 (10.1)	“Decreased risk of illness;”
		“Safest environment (home) for those who are immunocompromised;”
		“Safety regarding virus transmission.”
Increased flexibility	9 (13.0)	“More flexible scheduling;”
		“More flexibility for time/availability;”
		“Allow for a more flexible schedule for the client.”
Decreased travel time	16 (23.2)	“Allows clients to access services from remote locations without the client or therapist traveling several miles;”
		“More efficient/less tiring for therapist (not having to drive);”
		“Able to schedule sessions back to back without driving time between them and see more clients.”
Preferred modality for some	10 (14.4)	“Some individuals are interested and more engaged with technology;”
clients		“For some in the mental health population, engagement is increased;”
		“Privacy for families who do not wished to be known receiving services.”
Integration of technology	8 (11.6)	“Many of the goals of MT can be focused on virtually – with some adaptations and creativity!”
		“Able to share screen in order to share documents/videos.”

**TABLE 8:** Challenges of telehealth.

Category	No. (%)	Example quotations
Difficult with some	15 (21.7)	“Not all interventions are possible via telehealth;”
interventions/assessments		“Inability to do any music therapy techniques that require synchronously making music with the client;”
		“more challenging to complete assessments.”
Technical challenges	47 (68.1)	“Technical difficulties (internet, not having the proper equipment, etc.);”
		“Internet connection cutting out could possibly lead to sessions being canceled. Some clients have difficulty using technology so it is frustrating for them.”
Inability to provide	19 (27.5)	“Harder to do hand over hand techniques;”
physical cues		“Inability to touch, guide physically or offer emotional support when proximity is needed;”
		“Not able to offer physical support or stimulation (e.g., sometimes a simple prompt through touch is all someone needs to initiate action).”
Lack of therapist-client	27 (39.1)	“There may be trouble building therapeutic alliance;”
personal connection		“Potential feeling of a barrier. Not being able to read body language easily.”
		“More difficult to engage clients sometimes – no opportunity to directly interact using some interventions.”
Caregiver support challenges	15 (21.7)	“Caregivers/families don’t get the respite they normally get with in-home sessions.;”
		“A caregiver or assistant of some sort is needed on the other end of the call;”
		“Often other family members or persons in the background becoming a distraction.”
Decreased client engagement	18 (26.1)	“Some patients do not respond to the computer (recognizing it as interactive versus a movie);”
		“Children tend to tune out more easily, can’t be followed;”
		“Can be difficult to engage participants in remote setting, especially if they have cognitive impairments.”
Clients’ lack of resources	16 (23.2)	“My clients typically don’t have their own instruments;”
		“Not all individuals have access to internet/laptop;”
		“There are limitations without having in-person access to instruments and other manipulatives.”
Screen fatigue	5 (7.2)	“My clients. are experiencing screen fatigue from online school;”
		“Too much screen time.”
		“Increased attention requirements can cause earlier client fatigue.”

**TABLE 9:** Technology challenges.

Category	No. (%)	Example quotations
Internet connectivity	41 (59.4)	“Connection issues with internet are disruptive;”
		“Inconsistent quality of reception;”
		“Slow broadband speed;”
		“Low connectivity for clients in some areas.”
Lack of access to technology	8 (13.6)	“Client not having a laptop and using a phone;”
		“One family who didn’t have a webcam or microphone on their computer;”
		“Reaching families without video chat technology.”
Latency	36 (52.2)	“Sound lags when trying to make active music with clients;”
		“Latency and delay, not able to easily play together in sync or provide musical support in the same way as in a live setting;”
		“Lag in sound is the greatest difficulty.”
Poor audio quality	25 (36.2)	“Lack of quality speaker/microphone on the client’s end;”
		“Audio or visual quality is less than ideal;”
		“Sound quality and synchronous sound is not good.”
Poor visual quality	6 (8.7)	“Missing clear expressions from client;”
		“Choppy video.”
Unfamiliarity with technology	18 (26.1)	“Client caregiver unfamiliar with technology;”
		“Older patients sometimes are not used to computer and need help;”
		“Pediatric clients turning off audio/video without realizing.”
Zoom link confusion	4 (5.8)	“The first session always took about 15 min for families to get things connected correctly and signed in with the correct codes;”
		“Distributing the links correctly to clients, and them keeping them in a place for easy access.”
Lack of platform congruence	5 (7.2)	“Some student devices do not respond well in Zoom (i.e., Chromebooks);”
		“Not able to screen share or utilize necessary features on client-preferred platform.”
Difficult to see client	5 (7.2)	“Unable to point camera where therapist can see client;”
		“Limitations in what can be observed of patient response.”

## Discussion

### Associations Between NMT Practice and Telehealth Success

We found no association between the frequency of NMT practice and overall decrease in clinical hours from in-person to telehealth. However, correlation analysis found that NMT clinicians who used NMT techniques more frequently in their practice had fewer discontinued groups in the transition to telehealth and perceived themselves as likely to see clients over telehealth in the future (once COVID-19 is no longer a major threat). Likewise, therapists who stated that they found the NMT designation beneficial for telehealth used NMT more frequently for telehealth. A possible interpretation is that clinicians who used NMT techniques more frequently felt more prepared to implement these techniques over telehealth. Kern and Tague’s (2017) study provided evidence that music therapists who consistently employ the standardized techniques of NMT are more likely to have full time employment than those who practice forms of traditional music therapy. The current study examined the frequency of NMT technique usage within the NMT population without comparison to non-NMTs. Future research may compare NMTs and non-NMTs to ascertain whether having an NMT designation has advantages for job and client retention over telehealth. Beyond the potential benefits of using NMT techniques more exclusively, accessing support through global and local support meetings as well as informal conversations with colleagues may have contributed to NMTs’ successful transition to telehealth.

### Non-sensorimotor Techniques Are Transferable From In-Person to Telehealth

There were no significant differences regarding which NMT domains were used in-person compared to telehealth work. This indicates that overall, NMTs continued to address the same goals over telehealth as they had during in-person sessions. However, within the sensorimotor domain, there was a prominent reduction in the use of RAS to address lower sensorimotor and gait goals. RAS is an effective clinical technique for rehabilitation of gait function with supporting evidence from randomized control trials and studies in Parkinson’s disease (Cubo et al., 2004; Murgia et al., 2018; Thaut et al., 2019), stroke (Thaut et al., 2007; Cha et al., 2014; Mainka et al., 2018; Elsner et al., 2020), cerebral palsy (Kim et al., 2012; Wang et al., 2013), and multiple sclerosis (Seebacher et al., 2017). RAS is supported by numerous lines of fundamental neuroscience evidence, including a recent finding that RAS attenuates dopamine response in younger adults (Koshimori et al., 2019).

Our qualitative analysis revealed that NMTs had safety concerns associated with RAS (e.g., increased fall risk), since they were unable to provide physical support. Some NMTs continued to work on physical goals, such as gait and balance training with clients over telehealth, because their clients had a caregiver present who could support the client physically. The requisite for caregiver support in telehealth gait rehabilitation is consistent with protocols for other telehealth rehabilitation programs (outside of music therapy). In a physiotherapist-led gait-training intervention for adults at their homes, licensed practical nurses present in clients’ homes were trained to use specific hand placements to physically support clients during the intervention to prevent falls (Hoffman and Prieto, 2016). The need for caregiver support during telehealth to continue physical goals is a strong consideration for future implementation of telehealth. NMT sensorimotor techniques, like RAS, may be contraindicated for clients who do not have adequate physical support. Conversely, it is recommended that efforts be made to enable access to home support for clients who stand to benefit from RAS.

Although safety concerns for telehealth RAS should be addressed, there is evidence that telerehabilitation can be used to improve or maintain sensorimotor and gait function. A recent Cochrane systematic review investigated the effects of 22 Randomized Controlled Trials of telerehabilitation in stroke (compared to in-person rehabilitation and usual care) on numerous outcomes including activities of daily living, mobility, balance, and upper limb function. The synthesis concluded that there is low or moderate-level evidence that telerehabilitation is more or similarly effective to in-person rehabilitation or usual care (no rehabilitation) (Laver et al., 2020). In an intervention for persons with Parkinson’s disease, telerehabilitation (compared to usual care) was implemented following a 4-week in-person clinician-supported virtual reality program. Results revealed that the group that continued with the telerehabilitation program showed additional enhancements in upper motor ability, while usual care showed decreasing balance and mobility (Isernia et al., 2020). Telerehabilitation shows promising results for maintaining sensorimotor function and balance following an in-person program. Canadian Stroke Best Practices (Heart and Stroke Foundation of Canada, 2019) and U.S. Veterans Affairs/Department of Defense Clinical Practice Guidelines for the Management of Stroke Rehabilitation (The Management of Stroke Rehabilitation Work Group, 2019) recommend RAS for lower-limb gait training including gait velocity, cadence, stride length, and gait symmetry. Therefore, future studies are warranted to compare telehealth RAS, in-person RAS, and/or usual care.

Other than the decrease of RAS in the sensorimotor domain, NMTs continued to work on the same techniques in the cognitive and speech/language domains over telehealth and in-person sessions. The successful continuation of cognitive and speech/language interventions over telehealth was seen in previous research (Langbecker et al., 2019; Harkey et al., 2020). Periodically, the focus of techniques within domains shifted: seventy-nine percent of respondents in our qualitative analysis answered affirmatively to the question about whether therapists were addressing goals related to the stresses and mood dysregulations. Descriptive results from the quantitative questions regarding client goals implies that those who were already addressing some cognitive psychological goal [e.g., through music psychosocial training and counseling (MPC)] in in-person NMT sessions continued to do so online, even if the specific focus of these MPC interventions altered. Thus, despite the challenges of telehealth, clients continued to receive consistent services from their NMT therapists online. This was also reflected in the qualitative data: the second-most-common benefit of telehealth articulated by participants was continuation of services.

### Place of Employment Did Not Affect Telehealth Success

Private practice/NMT clinic owners, and employees of a private practice, NMT clinic, or a facility (institution, long-term care, school, etc.) had similar clinical hours. On average, NMTs had fewer clinical hours for telehealth sessions compared to in-person sessions. The decrease in hours overall from in-person to telehealth was expected: with the transition of services related to the onset of COVID-19, NMTs (on average) lost clinical hours regardless of employment setting. Although no interaction effect was found between employment setting and change in clinical hours, it is possible that some music therapy businesses were affected greatly by the pandemic and others less so. Our employment setting categories may not have been sensitive to changes in clinical hours dependent on population (i.e., schools versus long-term care in institutions) and other factors such as regional COVID-19 restrictions and funding constraints. Future studies may investigate the effect of regional differences (between countries, states, and provinces) and differences in health care infrastructure and insurance/public funding on NMT clinicians’ successful transition of services to telehealth.

### Benefits of Telehealth

It can be interpreted from the qualitative data that although in-person NMT may be superior to telehealth delivery in general, telehealth proved beneficial in many situations, despite its challenges. Accessibility was named as a major benefit of telehealth. 39% of respondents cited accessibility as a benefit of telehealth separate from the ability to continue sessions. The emergence of the theme of accessibility as distinct from the benefit of session continuation suggests that telehealth may be valuable as a continued platform to practice NMT beyond the pandemic so that remote clients can access services. As chronic neurological disorders become increasingly pervasive, especially amongst older populations, access to medical care becomes a challenge. The current study is certainly not the first to suggest that telehealth may help to solve the issue of providing care to remote clients: a study by Kim et al. (2017) found that a telemedicine program compared telehealth care to in-person care for individuals with dementia. They found that the cognitive decline in the patients who received telehealth care was comparable to those receiving in-person care (Kim et al., 2017; Schneider and Biglan, 2017). Harkey et al. (2020) found that for occupational therapists, physiotherapists, and speech-language pathologists, providing services to remote communities via telehealth greatly improved patient satisfaction with services. Limitations in accessibility typically occur as a result of isolation in remote and rural locations, disabilities, or impairments that impedes an individual’s ability to travel. The disproportionate geographical distribution of health-care professionals can be viewed as a dominant factor for limited access to health-care, especially in less developed countries (Schneider and Biglan, 2017). Telehealth, telemedicine, and telerehabilitation transgress geographical location boundaries and thus are an important future frontier for health care services such as NMT even after the pandemic has run its course (Vaudreuil et al., 2020).

Data from the present study indicates that participants believed certain clients may benefit particularly from a telehealth modality more than from in-person therapy. Previous studies suggested that some autistic clients may benefit from an online format more than an in-person format (Baker and Krout, 2009; Vaudreuil et al., 2020). Open-ended question responses regarding benefits of telehealth referred to the benefit of an online modality to clients with autism who normally struggled with attention, persons with aphasia, and individuals with mental health struggles. There were also clients who specifically did not benefit from telehealth. For example, one NMT reported that persons with cochlear implants did not find telehealth beneficial because of the much lower sound quality. These reports revealed a need for more studies focused on the benefits and disadvantages of telehealth NMT for specific client populations or specific goal areas. It is possible that while most individuals would benefit from in-person NMT, there are some for whom online or mixed online/in-person NMT may be helpful. Moreover, online NMT may be appropriate for clients who are unable to access in-person therapy.

Qualitative data also indicated that increased caregiver involvement aided the facilitation of NMT techniques requiring in-person support, improved the transfer of outcomes to daily life, and created opportunities for therapists to provide family-centered care. Open-ended question responses indicated that parent involvement in their children’s NMT sessions may have increased understanding of NMT goals and techniques. This increased understanding allowed parents to help pediatric clients to practice skills between sessions, thus speeding up the therapeutic process and improving transfer to real-life skills. Similarly, Thompson et al.’s (2014) found that family-centered music therapy yielded an increase in parent-perceived social interactions in autistic children outside of sessions. Some NMTs articulated that caregiver involvement allowed them to treat the entire family structure. Previous research has uncovered similar findings about the benefits of caregiver involvement in music therapy (Thompson et al., 2014). Benefits include improving relationships between parents and children (Ettenberger, 2017; Fuller and McLeod, 2019), helping children engage in sessions (Abad and Edwards, 2004), and addressing caregiver stress (Raglio et al., 2016). Having families and caregivers more present/involved in sessions sometimes posed challenges. NMTs reported difficulties when the client’s caregiver was unable to help with technology issues. Some respondents stated that family members talking in the background were distracting, and sometimes their presence created an impediment to client privacy. Despite these challenges, our analysis revealed caregiver support was often a requisite to continue with NMT over a telehealth platform. For example, several participants stated that when a caregiver was present, they were able to continue with sensorimotor interventions such as RAS over telehealth.

### Challenges of Telehealth

There are several distinct challenges to telehealth, not the least of which include a myriad of technical difficulties. Internet access, varying levels of comfort with technology, and audio/visual latency create numerous, often highly frustrating problems for therapists trying to provide quality service online. In-person group music making usually depends on the music-makers being in temporal synchrony with one another. There is a plethora of research on how being in temporal synchrony with one another during group-music making yields positive benefits such as increased empathy and prosocial behaviors (Hove and Risen, 2009). The inability to play in-time with clients limits non-verbal communication and therefore becomes a major drawback to telehealth. Live music is an important characteristic of in-person NMT techniques, especially those requiring synchronization of movements. This includes most sensorimotor techniques and some speech/language techniques. Unfortunately, the reality of internet and conferencing software limitations means that online telehealth platforms prevent live synchronized musical activity. Thus, even if technology is working perfectly, audio (and visual) latency prevents the interpersonal connection between client and therapist from being as intimate as when music-making happens live, in-person. For cognitive techniques, integration of multisensory cues and delayed feedback may increase the perceptual load and therefore present difficulties for selective attention (Cochrane et al., 2020). NMTs have adapted their intervention techniques by using more pre-recorded music and finding other creative ways to work around the lack of synchrony in telehealth. Although NMTs reported working with a range of platforms for NMT telehealth, all participants reported using Zoom. It is undetermined whether some technology challenges may have been specifically related to Zoom, or conversely whether Zoom resolved some technical issues. Future research should aim to determine how the lack of synchrony affects client progress. It may be that some techniques/goals are more impacted than others, depending on how dependent the technique is on synchronous music making.

Another important drawback to telehealth is the inability to provide physical cues to clients. Some NMT techniques rely specifically on physical guidance from the therapists (i.e., tapping a client’s hand during melodic intonation therapy (Thaut and Hoemberg, 2014). Sensorimotor interventions often require physical support to ensure clients’ safety. Not being able to provide these cues meant that NMTs had to rely exclusively on caregivers to provide this support, which meant providing caregivers required training during sessions. Clients without adequate caregiver support either had to take a break from working on certain goals, or in some cases stopped sessions altogether.

### Developing a Model for Neurologic Music Therapy via Telehealth

Telehealth offers an invaluable opportunity for therapists to provide NMT services that are accessible for the client and may increase the transfer of NMT gains to real world settings. Our data suggests that telehealth has the potential to be integrated into routine NMT services. The NMT Academy intends to use this data to develop strategies to navigate clinicians’ concerns and implement recommendations in training courses and global support meetings. Since frequent NMT usage was associated with positive perception of telehealth and success with group sessions, further trainings instructing how to translate NMT techniques effectively to telehealth may be beneficial for improving telehealth success. We recommend that future research examine when to apply telehealth as the sole method of delivery or in combination with in-person services, as well as to develop assessment tools to examine clients’ overall progress over telehealth compared with in-person sessions. Based on the current findings, below is the summary of the recommendations for the use of telehealth in NMT beyond the pandemic.

### Limitations

Although our survey was distributed to over 2,700 certified NMTs worldwide, our low response rate may be explained by the specific inclusion criteria for this study. Other professionals, such as physiotherapists and speech and language pathologists, who took the NMT training but were not accredited to practice music therapy in their region were excluded (see recruitment criteria in section “Data Collection”). Our criteria may have excluded therapists who continued working in-person with their clients, or who were not able to continue with any clients over telehealth. The greater proportion of North Americans (∼80%) may be attributed to the survey offered solely in English. Our sample included mostly female NMTs (∼90%), however, such distribution is consistent with the gender demographic of music therapists (Kern and Tague, 2017).

The heterogeneous client population of this cross-sectional survey study, inevitable differences in clinical experience, assessment and technique implementation methods, and self-report estimates made it difficult to assess client progress. The survey design meant that results were generalized to all clinical populations, with no indications of variability amongst groups. It is possible that certain clinical groups may have been impacted more acutely than others, such as older adults in long-term care homes which may have been locked down. Furthermore, one of our research questions concerned about the effectiveness of NMT versus non-NMT music therapy technique usage for the transition to telehealth. As we were sampling from the population of NMTs only, the only way to compare against non-NMT was to look at the frequency of NMT technique usage. Future studies may consider recruiting both NMTs and non-NMT music therapists to inform the use of music-based interventions via telehealth. Finally, our survey asked questions relating to the effects of the COVID-19 pandemic in a general manner, which meant that we were not able to capture the effects of lockdown versus no lockdown phase across different regions/countries.

**Table:** 

Recommendations for Telehealth in Neurologic Music Therapy	
**Integrate telehealth into routine care.**	Therapists should evaluate, on a case-by-case basis, whether clients would benefit from in-person NMT, telehealth, or a combination.
	Factors may include client location, client preference, presence/availability of caregivers.
**Mitigate safety concerns.**	When facilitating sensorimotor techniques (such as rhythmic auditory stimulation) online, therapists should consider when to implement physical support (i.e., a device or caregiver support).
	Have emergency contacts available for clients seen over telehealth in case of unforeseen safety challenges.
	Ensure therapy liability waivers and consent forms specify agreement to receive telehealth care.
**Identify clinical populations that could benefit from remote delivery.**	Clinicians should keep up-to-date on research regarding benefits of telehealth for specific populations and translate findings into their practice.
	The NMT Academy should keep a working bibliography of telehealth-related research publications for clinicians to access.
**Include client caregivers in NMT**	NMTs should actively educate caregivers on how to support clients during telehealth sessions.
**process over telehealth.**	NMTs should train caregivers to help with “homework” exercises to facilitate transfer and provide resources to this end (e.g., written description of exercises to practice, recorded music to use, etc.).
**Develop and share resources for telehealth NMT.**	NMTs should develop a repertoire of assessments that can be administered online and share these via the NMT support network.
	NMTs should continue to develop and share resources for implementing NMT techniques via technology.

## Conclusion

Upon the onset of the COVID-19 pandemic, many Neurologic Music Therapists (NMTs) used telehealth to continue to provide NMT services to clients when in-person service was not possible. Our main findings were: (1) The implementation of NMT techniques transferred well from in-person to telehealth all domains (sensorimotor, cognitive, and speech and language). NMTs’ adaptation strategies included increased caregiver involvement, the use of pre-recorded music, and reducing the implementation of sensorimotor techniques, such as RAS, when caregiver support was limited, (2) more frequent NMT technique usage by trained neurologic music therapists was advantageous for retaining group clients and was associated with a more positive view toward continuing telehealth in the future (i.e., once COVID-19 is no longer a major threat), (3) on average, NMTs working in all employment settings experienced a decrease in clinical hours over telehealth (i.e., after COVID-19) compared to the number of hours they had in-person (i.e., before COVID-19), (4) telehealth benefits include increased accessibility of NMT services, for therapists and clients who live in geographically remote and rural locations or have mobility or health concerns. We recommend that the benefits of caregiver involvement should be considered for in-person and telehealth NMT, (5) NMTs should continue to share resources to navigate the technological challenges of telehealth. Our findings support the notion that NMT can be delivered online and provides important considerations for adapting interventions and developing best practice guidelines for NMT telehealth. Some of our telehealth recommendations may be relevant to fields outside of NMT, as NMTs’ experiences of tele-therapy and rehabilitation may find resonance in the experiences of other music therapists and health professionals delivering care during the COVID-19 pandemic and beyond.

## Data Availability Statement

The datasets generated for this study are not readily available. Raw data cannot be shared publicly because of possible identifiable cases and Research Ethics Board restrictions for this project. Requests to access the datasets should be directed to the University of Toronto Research Ethics Board, ethics.review@utoronto.ca; or to CH-T, corene.thaut@mail.utoronto.ca.

## Ethics Statement

The studies involving human participants were reviewed and approved by University of Toronto Research Ethics Board. The participants provided their written informed consent to participate in this study.

## Author Contributions

All authors collaborated equally on devising the study, objectives, and survey design. MP wrote the first draft of the introduction. KK, NR, and TH wrote data collection and materials. TH and KK designed the quantitative analysis, conducted statistical analyses, and wrote the quantitative analysis methods and results. LC, MP, NR, and GT chose qualitative methods and conducted the qualitative analysis. All authors contributed to writing the discussion and conclusion. All authors contributed to revisions of the manuscript. CH-T supervised the project and provided feedback through all stages of the project and manuscript writing.

## Conflict of Interest

The authors declare that the research was conducted in the absence of any commercial or financial relationships that could be construed as a potential conflict of interest.
