# Supplementary material for: Neurologic Music Therapy via Telehealth: A Survey of Clinician Experiences, Trends, and Recommendations During the COVID-19 Pandemic
Source: Front Neurosci. 2021 Apr 8;15:648489. doi: 10.3389/fnins.2021.648489 (PMC8060694; doi:10.3389/fnins.2021.648489)
Supplement: Supplementary file 1 [file Data_Sheet_1.PDF]

NMT Telehealth Survey

Thank you for your participation!

Research Title: Challenges, benefits, and future directions for telehealth delivery of Neurologic Music Therapy: A cross-sectional survey.

Researchers:

PI: Dr. Corene Thaut

Co-investigators: Tara Henechowicz

Nicole Richard

Kyurim Kang

Lauren Cole

Gloria Tian

Marija Pranjic

Dear participants,

You are invited to participate in this survey from the Music and Health Science Research Collaboratory (MaHRC) at the University of Toronto. In this survey, we will ask for your perceptions of and experience with telehealth Neurologic Music Therapy to help develop best practice guidelines. This survey must be completed within two weeks from when you received this survey.

We are currently looking for participants who meet the following criteria:

Inclusion Criteria: please check the boxes to indicate that you meet the criteria
- Read and understand English
- Over the age of 18
- Active NMT affiliate, which means they have completed NMT training within the past 3 years or NMT fellowship within the past 5 years.
- Have practiced Music Therapy (including NMT or other techniques) over telehealth with at least one client or group.
- Must be permitted to practice Music Therapy in their region.

Exclusion Criteria:
- Lapsed NMT affiliates/training.
- Are not Music Therapists.

You must exclude yourself from participating in this study if you do not meet all of the inclusion criteria or if you meet any of the exclusion criteria.

Research Description:
This survey has two sections. The first set of questions aims to ascertain how COVID-19 has affected clinical practice to identify and disseminate best practice methods for online NMT clinical practice. The second section will be an opportunity for you to provide tips for post-secondary university students who may not have strong support on how they can use music to help cope during this challenging time. This will be used to create a multimedia music-for-coping tool for students.

Time Involvement: This survey will take approx 10 to 20 minutes.

Risk and Benefits:
Risk: This study poses minimal risk to participants. For example, it is possible that some questions might spark some thoughts related to uncomfortable experiences. However, your response will be optional and you can end your participation at any time.

Benefits: There are no personal benefits in participating in this study. However, your answers will contribute a better understanding of 1) neurological music therapy in the telehealth sessions 2) the effects of music on the well-being of college students during a pandemic.

Participant's Rights:
Your participation is entirely voluntary. You are free to discontinue participation at any time without penalty. You also have the right to refuse to answer particular questions. Due to the anonymity and confidentiality of this survey, you cannot withdraw your data after you have completed the survey.

Confidentiality:
All collected data is anonymous and confidential. Any contact information that you provide will be used only for the purpose of compensation and will be provided and stored separately from your responses. All survey data and any identifying information will be protected by this researcher through encryption and secure storage. Data collected in this study will be reported in research publications and/or conference presentations, but no private or identifying information will be published. We will destroy any identifying information after data collection and the draw for gift-card winners is completed. However, we will store the de-identified anonymous survey data on a secure server at the Neurologic Music Therapy Academy indefinitely.

Compensation:
All participants who complete section 1 of the survey can be entered in a random draw for 1 of 20 gift cards with a value of $20 CAD. If you complete both sections 1 and 2 of the survey, you can be entered in a second chance to win a gift card. If you withdraw from the study, you will not have an opportunity to enter the draw.

The research study you are participating in may be reviewed for quality assurance to make sure that the required laws and guidelines are followed. If chosen, (a) representative(s) of the Human Research Ethics Program (HREP) may access study-related data and/or consent materials as part of the review. All information accessed by the HREP will be upheld to the same level of confidentiality that has been stated by the research team.

If the participant wishes, they can contact the Research Oversight and Compliance Office – Human Research Ethics Program at ethics.review@utoronto.ca or 416-946-3272, if they have any questions about their rights as participants.

Contact information:
If you have any questions, concerns, or complaints about this research, its procedures, risks, and benefits, you can contact our team at **mahrctelehealth@gmail.com**

Signature of Research Participant:

* 1. By checking the boxes below, you are providing your electronic signature to participate in this study.

Your signature indicates that you have read and understood the information provided above and confirm and acknowledge the following

- [ ] I meet all of the inclusion criteria and do not hold any of the exclusion criteria; I am therefore are eligible to participate in the survey.
- [ ] I have had the opportunity to ask questions, and all of my questions have been answered to my satisfaction.
- [ ] I understand that my participation is voluntary.
- [ ] I understand that I may withdraw my consent and discontinue participation at any time during the survey without penalty.

NMT Telehealth Survey

Demographics

* 2. NMT designation

- NMT
- NMT-Fellow
- Lapsed NMT (i.e., have not taken training in 3+ years, or have not retaken fellowship training in 5+ years)
- Student affiliated member
- Other (please specify)

* 3. What are your professional credentials, which allow you to practice music therapy in your region (e.g., Music Therapist-Board Certified [MT-BC])?

4. Years of practicing **NMT**

0 20

5. Years of practicing music therapy/other professional service (PT/OT/SLP)

0 40

* 6. What is the highest education level you have completed?

- Bachelor's Degree
- Master’s Degree
- PhD
- Graduate Certificate
- Other (please specify)

* 7. In which country do you currently practice music therapy?

- United States
- Canada
- Other (please specify)

* 8. Gender

- Female
- Male
- Non-binary/third gender
- Prefer not to say
- Prefer to self-describe

9. Age (please type number in box below)

- Check this box if you prefer not to specify

Age

* 10. What is your current primary type of employment

- Currently not employed
- Own/run a private practice
- Subcontractor/Independent Contractor for private practice(s)
- Employed by a university or institute of higher learning as an educator
- Employed by a private practice
- Employed by a school board
- Employed by a healthcare facility (includes hospitals and rehabilitation centres)
- Employed in long-term care
- Employed in a correctional facility
- Student
- Intern
- Other (please specify)

11. How many clinical hours per week did you practice prior to the transition to telehealth?

0 100

12. How many clinical hours per week have you practiced since telehealth sessions began?

0 40

* 13. Do you consider your current caseload full-time?

* 14. With which populations do you work? Check all that apply.

- [ ] Neurodevelopmental populations (autism, developmental disabilities)
- [ ] Geriatric care and dementia
- [ ] Mental health/Psychiatric
- [ ] Acute Neurorehabilitation (speech/cognitive/motor)
- [ ] Chronic Neurorehabilitation (speech/cognitive/motor)
- [ ] Physical rehabilitation (Orthopaedics, sports injury, etc)
- [ ] Adapted Music education
- [ ] End of life/palliative
- [ ] Other (please specify)

* 15. Age groups served (check all that apply):

- [ ] Neonatal care
- [ ] Children
- [ ] Adolescents
- [ ] Adults
- [ ] Older adults

NMT Telehealth Survey

Perceptions

* 16. I support the concept and value of telehealth

- ○ Strongly agree
- ○ Agree
- ○ Neither agree nor disagree
- ○ Disagree
- ○ Strongly disagree

* 17. Please list any benefits that telehealth provides:

* 18. Please list any of the drawbacks to telehealth:

* 19. The NMT designation has been beneficial and/or had a direct positive impact on my ability to do telehealth

- ○ Strongly agree
- ○ Agree
- ○ Neither agree nor disagree
- ○ Disagree
- ○ Strongly disagree

* 20. What supports have helped you to successfully provide telehealth sessions (check all that apply)?

- ☐ Global NMT support meetings
- ☐ Local NMT support meetings
- ☐ Informal support conversations with colleagues
- ☐ Support from facility
- ☐ Other (please specify)

* 21. Do you see yourself continuing to use Telehealth in the future (i.e., after COVID is no longer a major threat)

- ◯ Not at all
- ◯ For some clients, perhaps
- ◯ Unsure
- ◯ Likely
- ◯ Yes, definitely

Technology

* 22. Which platform do you use to facilitate telehealth NMT sessions? (select all that apply)

- [ ] Zoom
- [ ] Telephone
- [ ] FaceTime
- [ ] Whatsapp Video
- [ ] Facebook Video
- [ ] Google hangouts
- [ ] doxy.me
- [ ] Other (please specify)

* 23. Briefly list the main technical difficulties you experienced

24. How many sessions did it take until the majority of technical issues were resolved?

0 20

Assessments

* 25. In general, I am able to carry out assessments over telehealth

- Strongly agree
- Agree
- Neither agree nor disagree
- Disagree
- Strongly disagree

* 26. I am able to carry out assessments over telehealth for

	Strongly agree	Agree	Neutral	Disagree	Strongly disagree	N/A
Gait						
Upper motor						
Psychosocial						
Attention						
Executive function						
Memory						
Speech/Language						

* 27. I used the following methods to collect assessment information during my **in-person** sessions (select all that apply)

- [ ] Standardized non-self report assessment tools
- [ ] Standardized self-report assessment tools
- [ ] Facility specific non-standardized assessment tools
- [ ] Client-specific non-standardized assessment tools
- [ ] Observing client behavior (non-standardized)
- [ ] Diagnostic assessments performed by other health care practitioners
- [ ] Standardized assessments performed by other health care practitioners
- [ ] Interviews with caregivers
- [ ] Interviews with clients
- [ ] Online Assessment tools
- [ ] Other (please specify)

* 28. I used the following methods to collect assessment information during my **telehealth** sessions (select all that apply)

- [ ] Standardized non-self report assessment tools
- [ ] Standardized self-report assessment tools
- [ ] Facility specific non-standardized assessment tools
- [ ] Client-specific non-standardized assessment tools
- [ ] Observing client behavior (non-standardized)
- [ ] Diagnostic assessments performed by other health care practitioners
- [ ] Standardized assessments performed by other health care practitioners
- [ ] Interviews with caregivers
- [ ] Interviews with clients
- [ ] Online Assessment tools
- [ ] Other (please specify)

Clinical Practices

Session Length and Frequency

* 29. Prior to COVID-19, I conducted sessions

- ◯ In person
- ◯ Through telehealth
- ◯ Both in person and through telehealth

* 30. Since adjusting to COVID-19, I conducted sessions

- ◯ In person
- ◯ Through telehealth
- ◯ Both in person and through telehealth

31. What percentage of your previously in-person **individual clients** were discontinued since telehealth session was implemented (%)?

0 100

32. What percentage of your previously in-person **groups** were discontinued since telehealth session was implemented (%)?

0 100

33. How many new **individual clients** have begun services with you over telehealth?

0 50

* 34. How many new **groups** have begun services with you over telehealth?

0 50

All following questions pertain to both individual and/or group sessions

* 35. How frequently do you use NMT?

	Always	Usually	Sometimes	Rarely	Never
During in-person sessions	○	○	○	○	○
During telehealth sessions	○	○	○	○	○

* 36. How frequently do you use non-NMT music therapy techniques?

	Always	Usually	Sometimes	Rarely	Never
During in-person sessions	○	○	○	○	○
During telehealth sessions	○	○	○	○	○

Clinical Practice

Goals

* 37. What goal areas were you addressing during **in-person** sessions (select all that apply)?

- [ ] Lower motor goals (e.g., gait)
- [ ] Upper motor goals
- [ ] Speech/Language goals
- [ ] Psychosocial goals
- [ ] Attention skills
- [ ] Executive functioning goals
- [ ] Memory goals
- [ ] Other (please specify)

* 38. What goal areas were you addressing during **telehealth** sessions (select all that apply)?

- [ ] Lower motor goals (e.g., gait)
- [ ] Upper motor goals
- [ ] Speech/Language goals
- [ ] Psychosocial goals
- [ ] Attention skills
- [ ] Executive functioning goals
- [ ] Memory goals
- [ ] Other (please specify)

* 39. Have you addressed goal areas related to stresses, isolation, disruption to everyday routines, and other challenges caused by the pandemic? Please explain:

NMT Telehealth Survey

Clinical Practice

NMT Techniques

* 40. In the **motor** domain, which NMT techniques do/did you use _________

	Rhythmic Auditory Stimulation (RAS)	Patterned Sensory Enhancement (PSE)	Therapeutic Instrumental Music Performance (TIMP)	N/A
**In person**	☐	☐	☐	☐
**Telehealth**	☐	☐	☐	☐

* 41. In the **cognitive** domain, which NMT techniques do/did you use _________

	Music Attention Control Training (MACT)	Music Executive Functioning Training (MEFT)	Associative Music and Memory Training (AMMT)	Musical Sensory Orientation Training (MSOT)	Musical Echoic Memory Training (MEM)	Auditory Perception Training (APT)	Auditory Perception Training: Sensory Integration (APT:SI)	Musical Mnemonics Training (MMT)	Musical Neglect Training (MNT)	Music in Psychosocial Training and Counselling (MPC)	N/A
**In person**	☐	☐	☐	☐	☐	☐	☐	☐	☐	☐	☐
**Telehealth**	☐	☐	☐	☐	☐	☐	☐	☐	☐	☐	☐

* 42. In the **cognitive** domain **within MPC** (music psychosocial training and counselling), which areas do/did you address _________

	Social Competence Training	Mood Vectoring	Cognitive Reorientation	Affect Modification	Behaviour Modification	N/A
**In person**	☐	☐	☐	☐	☐	☐
**Telehealth**	☐	☐	☐	☐	☐	☐

Other (please specify)

* 43. In the **speech** domain, which NMT techniques do/did you use _________

	Developmental Speech and Language Training Through Music (DSLM)	Musical Speech Stimulation (MUSTIM)	Symbolic Communication Training Through Music (SYCOM)	Melodic Intonation Therapy (MIT)	Rhythmic Speech Cueing (RSC)	Oral Motor and Respiratory Exercises (OMREX)	Vocal Intonation Therapy (VIT)	Therapeutic Singing (TS)	N/A
**In person**	☐	☐	☐	☐	☐	☐	☐	☐	☐
**Telehealth**	☐	☐	☐	☐	☐	☐	☐	☐	☐

NMT Telehealth Survey

Clinical Practice

Implementation

* 44. I have successfully used **live music** during

	Strongly agree	Agree	Neither agree nor disagree	Disagree	Strongly disagree
**In-person**	◯	◯	◯	◯	◯
**Telehealth**	◯	◯	◯	◯	◯

* 45. I have successfully used **pre-recorded music** during

	Strongly agree	Agree	Neither agree nor disagree	Disagree	Strongly disagree
**In-person**	◯	◯	◯	◯	◯
**Telehealth**	◯	◯	◯	◯	◯

* 46. I have strategies for successful sessions when clients have limited access to musical instruments.

◯ Strongly agree

◯ Agree

◯ Neither agree nor disagree

◯ Disagree

◯ Strongly disagree

Safety

* 47. Are there any NMT techniques that you have client safety concerns for administering through telehealth?

Explain your concerns:

* 48. How have you addressed safety concerns for clients during telehealth sessions?

- [ ] Increased caregiver involvement in sessions
- [ ] Avoided doing interventions that would require in-person **physical** support
- [ ] Avoided doing interventions that would require in-person **emotional** support
- [ ] Other (please specify)

Caregiver Involvement

* 49. Caregivers have been involved during:

	Always	Usually	Sometimes	Rarely	Never
In person	○	○	○	○	○
Telehealth	○	○	○	○	○

* 50. I feel that caregiver involvement is beneficial for a telehealth model

- ○ Strongly agree
- ○ Agree
- ○ Neither agree nor disagree
- ○ Disagree
- ○ Strongly disagree

Please briefly explain:

NMT Telehealth Survey

Draw Entry for Completing Section 1

Thank you for participating in this survey! If you would like to be entered to win one of twenty gift cards valued at $20 CAD, please send an email to mahrctelehealth@gmail.com with subject heading "Draw Entry." Your email address will not be connected to your particular survey answers. If you do not send an email to the address specified, you will not be entered into the draw.

NMT Telehealth Survey

Section II

The survey is complete! However, if you want to receive an additional entry to win a $20.00 CAD gift card, we invite you to complete the following short (two open-ended questions) survey on music and self-care strategies for students.

Many post-secondary students have experienced challenges due to the sudden changes and isolation in reaction to the pandemic, with potentially limited physical or financial access to telehealth services. Our aim with this set of questions is to gather suggestions to create a music-for-coping toolkit providing suggestions to enhance wellness in students in the current situation. We will synthesize the responses to create our multimedia toolkit for students, delivered via social media clips, videos, and blog posts.

As Neurologic Music Therapists who have helped clients cope with similar challenges, we invite you to share any advice on how to use music for wellness for students. We intend to suggest ideas for students on how to use music in their everyday lives to enhance wellness in a general manner. We disclaim that this program is not an intervention and we will not provide clinical advice, treatment, or mental health recommendations. We will encourage students to seek medical help if needed.

51. Due to the disruptions of the COVID-19 pandemic, many post-secondary students have experienced stress, anxiety, uncertainty, isolation, loneliness, sleep issues, and so on. In general, what recommendations do you have for post-secondary students to use music to cope and improve wellness when faced with challenges like these?

52. Many students have experienced disruptions and changes in routines such as increased remote working and online learning. How would you recommend that students use music to enhance work ethic, concentration and productivity?

NMT Telehealth Survey

Draw Entry

If you filled out section two, and would like to receive an additional entry into the draw for a $20 CAD gift card, please send an email to mahrctelehealth@gmail.com with the subject line "Draw Entry."
